# Bone regeneration in inflammation with aging and cell-based immunomodulatory therapy

**DOI:** 10.1186/s41232-023-00279-1

**Published:** 2023-05-25

**Authors:** Junichi Kushioka, Simon Kwoon-Ho Chow, Masakazu Toya, Masanori Tsubosaka, Huaishuang Shen, Qi Gao, Xueping Li, Ning Zhang, Stuart B. Goodman

**Affiliations:** Department of Orthopaedic Surgery, Stanford University School of Medicine, Stanford, CA USA

**Keywords:** Bone regeneration, Inflammation, Aging, Inflammaging, Mesenchymal stem cell, Cell therapy, Immunomodulation, Macrophage, Exosome

## Abstract

Aging of the global population increases the incidence of osteoporosis and associated fragility fractures, significantly impacting patient quality of life and healthcare costs. The acute inflammatory reaction is essential to initiate healing after injury. However, aging is associated with “inflammaging”, referring to the presence of systemic low-level chronic inflammation. Chronic inflammation impairs the initiation of bone regeneration in elderly patients. This review examines current knowledge of the bone regeneration process and potential immunomodulatory therapies to facilitate bone healing in inflammaging.

Aged macrophages show increased sensitivity and responsiveness to inflammatory signals. While M1 macrophages are activated during the acute inflammatory response, proper resolution of the inflammatory phase involves repolarizing pro-inflammatory M1 macrophages to an anti-inflammatory M2 phenotype associated with tissue regeneration. In aging, persistent chronic inflammation resulting from the failure of M1 to M2 repolarization leads to increased osteoclast activation and decreased osteoblast formation, thus increasing bone resorption and decreasing bone formation during healing.

Inflammaging can impair the ability of stem cells to support bone regeneration and contributes to the decline in bone mass and strength that occurs with aging. Therefore, modulating inflammaging is a promising approach for improving bone health in the aging population. Mesenchymal stem cells (MSCs) possess immunomodulatory properties that may benefit bone regeneration in inflammation. Preconditioning MSCs with pro-inflammatory cytokines affects MSCs’ secretory profile and osteogenic ability. MSCs cultured under hypoxic conditions show increased proliferation rates and secretion of growth factors. Resolution of inflammation via local delivery of anti-inflammatory cytokines is also a potential therapy for bone regeneration in inflammaging. Scaffolds containing anti-inflammatory cytokines, unaltered MSCs, and genetically modified MSCs can also have therapeutic potential. MSC exosomes can increase the migration of MSCs to the fracture site and enhance osteogenic differentiation and angiogenesis.

In conclusion, inflammaging can impair the proper initiation of bone regeneration in the elderly. Modulating inflammaging is a promising approach for improving compromised bone healing in the aging population.

## Background

Bone regeneration is essential for treating acute fractures, bone defects associated with non-unions, infection, tumors, and in conditions such as osteoporosis. Fractures increase significantly with age and are more challenging in elderly patients [1]. With aging, the balance between removing old bone and forming new bone is disrupted, resulting in osteoporosis [2]. Fractures can have severe consequences, including decreased mobility and independence, prolonged hospitalization, and even death. The global aging of the population has led to an increase in the incidence of osteoporosis and associated fragility fractures, which significantly impact patient quality of life and healthcare costs [3]. Therefore, it is essential to understand the mechanisms underlying bone fragility and how bone regeneration changes as the population ages.

Bone regeneration is a complex process that involves multiple stages, including inflammation, repair, and remodeling [4]. Appropriate regulation of the acute inflammatory reaction is essential to initiate healing after injury. However, aging is associated with ‘inflammaging,’ which refers to a low baseline level of chronic systemic inflammation without an apparent infection or other specific cause. Inflammaging increases the risk of age-related diseases and functional decline [5, 6]. Consequences of chronic inflammation include changes in the immune system and underlying medical conditions such as osteoporosis, diabetes, and cardiovascular disease. Uncontrolled chronic inflammation can impair the proper initiation of bone regeneration in the elderly. Ameliorating chronic inflammation and appropriate modulation of the inflammatory response are potential therapeutic targets for improving bone regeneration in these patient groups [7]. Therefore, understanding the role of inflammation in bone healing in the aging population is crucial for developing effective treatments for fractures and bone defects in this population.

In this review, we examine the current knowledge of the bone regeneration process and immunomodulatory therapy for bone healing in inflammaging. We also discuss the different types of cell-based immunomodulatory therapies that have been investigated, the mechanisms by which the cell-based therapies promote bone regeneration, and the challenges that remain to be addressed to optimize the therapy for bone regeneration in inflammaging.

## Bone regeneration and the acute inflammatory response

### Acute inflammation after fracture

Inflammation is a critical component of the healing process after a fracture. An acute injury damages the local bone, blood vessels, and soft tissues and triggers tissue-resident macrophages and other local immune cells to initiate the inflammatory cascade. This acute inflammatory phase lasts about 3 days in mice, 4 days in rats, and 1 week in humans [8, 9].

**Fig. 1:**
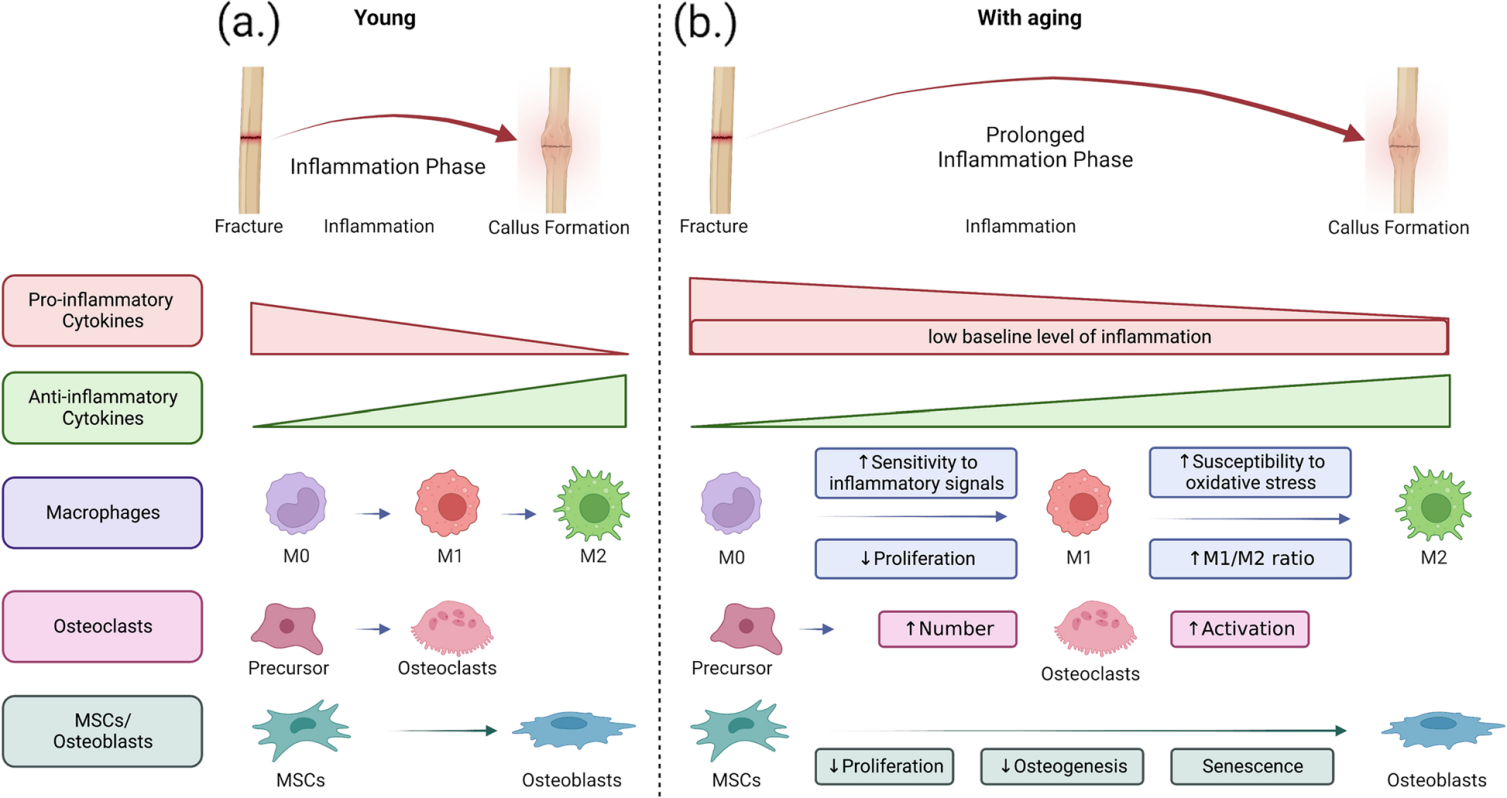
The differences in the acute inflammatory phase after fracture between the young and aging state. **a** After a bone fracture, the acute inflammatory phase is initiated, resulting in the formation of a hematoma at the fracture site and the infiltration of mobilized polymorphonuclear neutrophils (PMNs) to clear dead cells and debris. PMNs secrete pro-inflammatory chemokines, which attract macrophages, stem cells, and other progenitor cells to the site of injury to facilitate repair. Macrophages (M0) can be activated into two major subtypes: pro-inflammatory M1 and anti-inflammatory M2 macrophages. Initially, macrophages exhibit an M1 phenotype, but as the healing process progresses, they polarize toward an M2 phenotype. This shift is associated with a decrease in pro-inflammatory cytokines and an increase in anti-inflammatory cytokines. As the levels of anti-inflammatory cytokines rise, mesenchymal stem cells (MSCs) differentiate into osteoblasts, promoting new bone formation, while osteoclasts break down the damaged bone tissue. This coordinated process allows for the remodeling of the bone, ultimately leading to the restoration of bone structure and function. **b** Aging is associated with a persistent, low-grade, subclinical systemic inflammatory state, as evidenced by elevated circulating pro-inflammatory cytokines at baseline. The persistence of high expression of inflammatory cytokines prolongs the inflammation phase. Aged macrophages show increased sensitivity and responsiveness to inflammatory signals, increased susceptibility to oxidative stress, and lower proliferation. The persistent chronic inflammation that results from the failure to repolarize macrophages from the M1 to M2 phenotype leads to increased osteoclast activation and decreased osteoblast formation, resulting in increased bone resorption and decreased bone formation during healing. In aging, the decreased number and proliferative capacity of MSCs further contribute to impaired bone healing. Aged MSCs are more likely to become senescent and have lower osteogenic potential. Thus, the interplay between macrophages, osteoclasts, and MSCs is altered with aging, leading to impaired bone healing

During this phase, a hematoma forms a scaffold at the fracture site. This is accompanied by the invasion of mobilized polymorphonuclear neutrophils (PMNs) for the removal of dead cells and debris, secretion of pro-inflammatory chemokines, such as interleukin (IL)-1, IL-6, tumor necrosis factor-alpha (TNF-α), macrophage colony-stimulating factor (M-CSF), and inducible nitric oxide synthase (iNOS) to further mobilize macrophages [8, 10]. Although PMNs are essential in the early stages of inflammation, prolonged activation of PMNs is detrimental to fracture healing [10]. TNF-α receptors (p55 and p75) double knockout mice show impaired intramembranous bone formation and reduced mRNA expression of type 1 collagen and osteocalcin [11]. Inhibition of the C–C motif chemokine ligand 2 (CCL2)/CC-chemokine receptor 2 (CCR2) axis also impairs inflammation and bone regeneration [12]. Thus, suppression of the inflammatory response impairs fracture healing. Thereafter, cytokines and inflammatory mediators released by macrophages further attract stem cells and other progenitor cells to the fracture site to coordinate the repair process. Prolonged inflammation inhibits this step and increases the risk of complications such as non-union [13]. Thus, an acute inflammatory response is necessary for fracture healing, but a prolonged inflammatory response inhibits fracture healing. Figure 1a summarizes the acute inflammatory phase after a fracture.

### The role of the macrophage in bone repair

Macrophages contribute significantly to both the innate and adaptive immune systems, thereby maintaining physiological homeostasis [14]. Macrophages are important in bone formation at the physiological state and during bone repair [14–16]. A macrophage Fas-induced apoptosis transgenic model demonstrated that bone marrow macrophages mediate parathyroid hormone-dependent bone regeneration [17].

Macrophages also secrete numerous cytokines, growth factors, and chemokines during the inflammatory phase of bone healing, including, bone morphogenetic proteins (BMP), transforming growth factor-beta (TGF-β), insulin-like growth factor (IGF), fibroblast growth factor (FGF), and platelet-derived growth factor (PDGF) [11, 18–20]. Macrophages also secrete chemokines such as CCL2 and macrophage inflammatory protein-1 (MIP-1), which are essential for mesenchymal stem cells (MSCs) homing and migration to the site of injury [21].

Macrophages can be broadly divided into two major subtypes based on their activation status: M1 and M2 macrophages [22]. M1 macrophages can be activated by interferon-gamma (IFN-γ) and lipopolysaccharides (LPS), and are characterized by their pro-inflammatory properties, which promote bone resorption. On the other hand, M2 macrophages can be activated by IL-4 or IL-13 and are characterized by their anti-inflammatory properties, which promote bone formation and contribute to bone regeneration [22]. During the healing process, macrophages initially exhibit an M1 phenotype and shift to an M2 phenotype [23, 24] that is mediated by both autocrine signaling and paracrine signaling from other cells at the fracture site, including MSCs [23, 24]. In humans and other species, this original two-pronged macrophage classification has proved overly simplistic; newer techniques such as flow and mass cytometry and single-cell RNA sequencing have identified macrophage phenotype as a spectrum reflecting the local biological milieu [25, 26].

Acute inflammation is essential for fracture repair because acute inflammation stimulates angiogenesis and promotes MSCs proliferation and differentiation into osteoblasts [27]. Precise polarization of M1 and M2 macrophages at 72 or 96 h after co-culture enhances this effect [28]. Additionally, M2 macrophages survive longer than M1 macrophages, highlighting the transient and early role of M1 macrophages in bone formation [29].

### MSCs are essential for bone repair

MSCs are essential for bone regeneration because MSCs can differentiate into various cell types, including chondrocytes for endochondral ossification and osteoblasts for intramembranous ossification [21]. MSCs are also involved in the recruitment of macrophages during fracture healing [30, 31]. An essential step in bone regeneration is the localization of MSCs to the injury site. For example, the stromal cell-derived factor-1/C-X-C chemokine receptor 4 (SDF-1/CXCR4) ligand-receptor axis is critical for homing progenitor cells involved in fracture healing, as demonstrated by a parabiosis model [32] and murine allograft and autograft models [33]. However, the source of MSCs directly involved in fracture healing is still controversial. Some studies suggest that the periosteum and endosteum are essential sources of MSCs; others suggest that circulating cells directly contribute to d bone repair [34, 35]. MSCs and osteoprogenitors that migrate throughout the body may also contribute to subsequent bone regeneration during the bone repair process through their paracrine role [35–37].

## Bone regeneration in inflammaging

### Bone regeneration in aging

Bone regeneration in aging is a complex process influenced by multiple factors, including systemic and local signaling molecules, osteogenic and resorptive cells, immune cells, and blood microcirculation [38]. Studies in a mouse model of femoral fracture have shown that aging can negatively impact bone healing [39]. Aged mice displayed a weaker healing response characterized by decreased amount of callus, decreased bone density, less total cartilage and less bone content compared with younger mice [39]. Aging has also been associated with decreased numbers of osteoblasts and increased numbers of osteoclasts in a mouse model of rib fracture [40]. Adequate blood flow is essential for bone repair. Aging is associated with reduced local blood flow in bone, possibly due to impaired nitric oxide synthase pathways and reduced endothelium-dependent vasodilation [41]. In aged rats, femoral blood flow in the metaphyseal medulla is reduced by 45% [41].

### Inflammaging and aged macrophages

Aging is associated with a persistent, low-grade, subclinical systemic inflammatory state, as evidenced by elevated circulating proinflammatory cytokines [42, 43]. This state referred to as “inflammaging,” is characterized by increased pro-inflammatory, activated monocytes at baseline in aged mice [44]. Dysregulated chronic inflammation in aging tissues may disrupt the proper inflammation-mediated initiation of fracture healing in the elderly. In response to femur fractures, aged mice have a higher percentage of activated monocytes than younger mice. However, the mice do not show a concomitant increase in non-classical monocyte activation, which is characterized by the upregulation of genes involved in phagocytosis and tissue repair, leading to a pro-resolving and anti-inflammatory phenotype [44]. Inflammatory genes were downregulated in young fracture callus specimens 2 weeks after the fracture but remained elevated in older specimens [44].

Macrophages are central mediators of the inflammatory response. Aged macrophages show increased sensitivity and responsiveness to inflammatory signals. While M1 macrophages are activated during the acute inflammatory response, proper resolution of the inflammatory phase involves polarizing pro-inflammatory M1 macrophages to the alternatively activated anti-inflammatory M2 phenotype more closely associated with tissue regeneration [45, 46]. However, in aging, the persistent chronic inflammation that results from the failure to polarize macrophages from the M1 to M2 phenotype leads to increased osteoclast activation and decreased osteoblast formation, resulting in increased bone resorption and decreased bone formation during healing [45]. Persistent unopposed inflammatory stimuli, such as TNF-α, are elevated at low levels during aging, promoting osteoclastogenesis and bone resorption [47].

Aged macrophages produce more nitric oxide under resting conditions and are more susceptible to oxidation [48]. When challenged with IFN-γ or LPS, aged macrophages increase the production of TNF-α, iNOS, IL-1β, and IFN-γ [49–51]. These findings suggest that aged macrophages maintain a pre-activated resting state that enhances their response to inflammatory stimuli. However, aged macrophages also show decreased phagocytic activity, nitrite bursting capacity, and autophagy [43].

Telomere shortening in aged macrophages contributes to macrophages’ increased susceptibility to oxidative stress and decreased granulocyte–macrophage colony-stimulating factor (GM-CSF)-dependent proliferation [52]. Loss of telomeres decreases signal transducer and activator of transcription 5a (STAT5a) oxidation and phosphorylation, ultimately suppressing GM-CSF-dependent macrophage proliferation [52]. Increased levels of S-endoglin, a transmembrane glycoprotein associated with inflammatory processes, decreases macrophage proliferation, reduces survival response to GM-CSF, increases oxidative stress, and compromises the function of aged macrophages [53]. Aged macrophages also have decreased DNA binding activity in the promoter region of the IAβ gene and decreased expression of the major histocompatibility complex (MHC) class II molecules [54].

### Impaired osteogenesis by aged mesenchymal stem cells

Aging is associated with the decreased number and proliferative capacity of MSCs in the bone marrow. This decline has been observed in rats and humans [55, 56]. The number of precursor cells and degree of proliferation in the iliac crest of healthy participants decreases markedly with age [56]. The number and proliferative capacity of MSCs harvested in older humans decreased [57]. The total number of nucleated cells in bone marrow aspirate also decreases with age, regardless of gender [58]. However, there is a gender difference in the decrease in the number of osteoblast progenitor cells, with a significant decrease in females but not males [58]. These data suggest that aging decreases the availability and proliferative capacity of MSCs for osteogenesis, and these changes may be dependent on the gender of the host.

Human MSCs (hMSCs) from elderly individuals have lower proliferative and osteogenic potential than hMSCs from younger patients [59, 60]. This impaired osteogenic potential is evident in the decreased number of colony-forming-unit alkaline phosphatase-positive (CFU-ALP +) cells in hMSCs from the elderly [59, 60]. In addition, hMSCs from the elderly have significantly shorter mean telomere restriction fragments, which may contribute to difficulty for hMSCs undergoing osteogenic differentiation [59]. Telomerase knockout MSCs (mTR^−/−^ MSCs) also fail to differentiate into chondrocytes and undergo early morphological changes [61]. Osteoblast differentiation is inhibited in a mouse model of Werner’s syndrome (premature aging) with shortened telomere length [62]. These findings suggest that telomere length may contribute to impaired osteogenesis in aged MSCs.

Chronic inflammation in tissues during aging is characterized by cytokines that promote cell senescence, known as the senescence-associated secretory phenotype (SASP) [63]. Cell cycle regulators, such as p16INK4A (interfere with CDK4 and CDK6 cell cycle kinases), are crucial in controlling cellular senescence and are often overexpressed in aged hMSCs [64]. As a result, aged hMSCs have increased numbers of senescence-associated β-galactosidase (SA-β-gal)-positive cells and apoptotic cells [65]. Aged hMSCs have a genetic defect in which *p53* and its targets *p21* and *BAX* (apoptosis regulator) genes are overexpressed [65]. Recent research in mice has supported the existence of SASP in the skeletal environment, which leads to senescence and impaired function in resident stem cells [66]. Cytokines such as TNF-α, IL-1, and IL-6 have been identified as mediators of this effect. Proinflammatory cytokines signal through the inflammatory mediator nuclear factor kappa-light-chain-enhancer of activated B cells (NFκB) activated in stem cells from aged mice. Further experiments have demonstrated that the pathologic activation of the NFκB in mouse skeletal stem and progenitor cells leads to cellular senescence and impaired osteogenic stem cell differentiation [67]. The elevated secretion of pro-inflammatory cytokines by senescent MSCs contributes to the age-related decline in bone regeneration by promoting inflammation and tissue remodeling, which can lead to bone loss and impaired bone repair.

The periosteum, a reservoir of MSCs known as periosteum-derived progenitor cells (PDPCs), is essential in bone healing [18, 68]. PDPCs, which reside in the inner layers of the periosteum, have a key role in endogenous bone repair and remodeling [34, 69]. A study comparing PDPCs from human donors of different ages found significant changes in aged PDPCs, including decreased expression of cell cycle proteins (Ki67 and p53), increased oxidative damage, and higher nitric oxide production [70]. In addition, the aged sample had significantly increased IL-6 mRNA and higher ratios of RANKL and osteoprotegerin (OPG), indicating a milieu favoring bone resorption [70]. A separate study evaluating the periosteal properties in the mandibles of young and aged pigs also found that the aged animals had a thinner periosteum, fewer type III collagen fibers, were more prone to calcification and stiffness and had impaired functional properties [71]. These findings suggest that aging negatively affects periosteal stem cells and their ability to support bone healing. Figure 1b summarizes the acute inflammatory phase after fracture with aging, highlighting the differences between the young and the aged state.

## Future therapy for bone regeneration by modulating inflammation

Inflammaging has been identified as a potential therapeutic target for bone repair in the elderly. As noted previously, inflammaging can impair the ability of stem cells, such as MSCs, to support bone regeneration and contribute to the decline in bone mass and strength that occurs with aging. Therefore, modulating inflammaging is a promising approach for improving bone regeneration in the aging population.

### Immunomodulatory properties of MSCs therapy

MSCs possess immunomodulatory properties that may benefit bone regeneration in inflammation [72, 73]. MSCs can modulate adaptive and innate immune responses through paracrine and juxtacrine signaling with immune cells [74]. In co-culture experiments, MSCs significantly reduced the production of pro-inflammatory cytokines (such as TNF-α, IL-1β, and IL-6) induced by LPS in murine macrophages. MSCs were associated with increased secretion of IL-10 by murine macrophages [75]. The ability of MSCs to suppress inflammatory activation in macrophages has also been demonstrated in an in vivo murine model, in which the administration of MSCs protected against LPS-induced septic shock [75]. This protective effect was lost after macrophage depletion or IL-10 inhibition, indicating that macrophages are the primary target of MSC-mediated immunomodulation [75]. MSCs reduce M1 macrophage polarization and induce M2 polarization in co-culture with macrophages through cytokines such as PGE2 and Il-10 [45, 76, 77]. In the co-culture of MSCs and macrophages, a significant upregulation of pro-inflammatory cytokines (such as IL-6, and TNF-α) was observed in M1 macrophages, while upregulation of growth factors, including TGF-β, VEGF, and IGF-1, was observed in M2 macrophages [78].

In addition to directly modulating macrophages, MSCs also regulate macrophage chemotaxis; macrophage recruitment is critical for the immune modulation mediated by MSCs. Human and murine bone marrow-derived MSCs secrete several important chemokines, including C–C motif chemokine ligand 2 (CCL2) and CCL4, potent chemoattractants for monocytes and macrophages [79, 80]. This MSC-mediated macrophage recruitment and macrophage phenotype modulation may enhance tissue regeneration [81]. One study found that the partial differentiation of MSCs to osteoblasts in vitro, followed by their implantation in a murine cranial defect model, led to the recruitment of macrophages and improved defect healing [82]. Local delivery of MSCs during the acute inflammatory stage has also enhanced bone healing in a murine long bone critical size defect model [83]. High-dimensional mass cytometry has further revealed the differences in cell composition, stem cell functionality, and immunomodulatory activity between bone graft transplantation and MSCs therapy in the murine bone defect model [84]. The study observed the active recruitment of multiple cell types, including MSCs and other immune cells, to the bone defect sites during the healing process [84]. MSCs can suppress adaptive immune responses by inhibiting the proliferation of CD4 + (“helper”) and CD8 + (“cytotoxic”) T cells and promote the expansion and immune suppressive potency of regulatory T cells (T-reg) through the secretion of cytokines, such as IL-10, TGF-β, PGE2, and HLA-G [85].

**Fig. 2:**
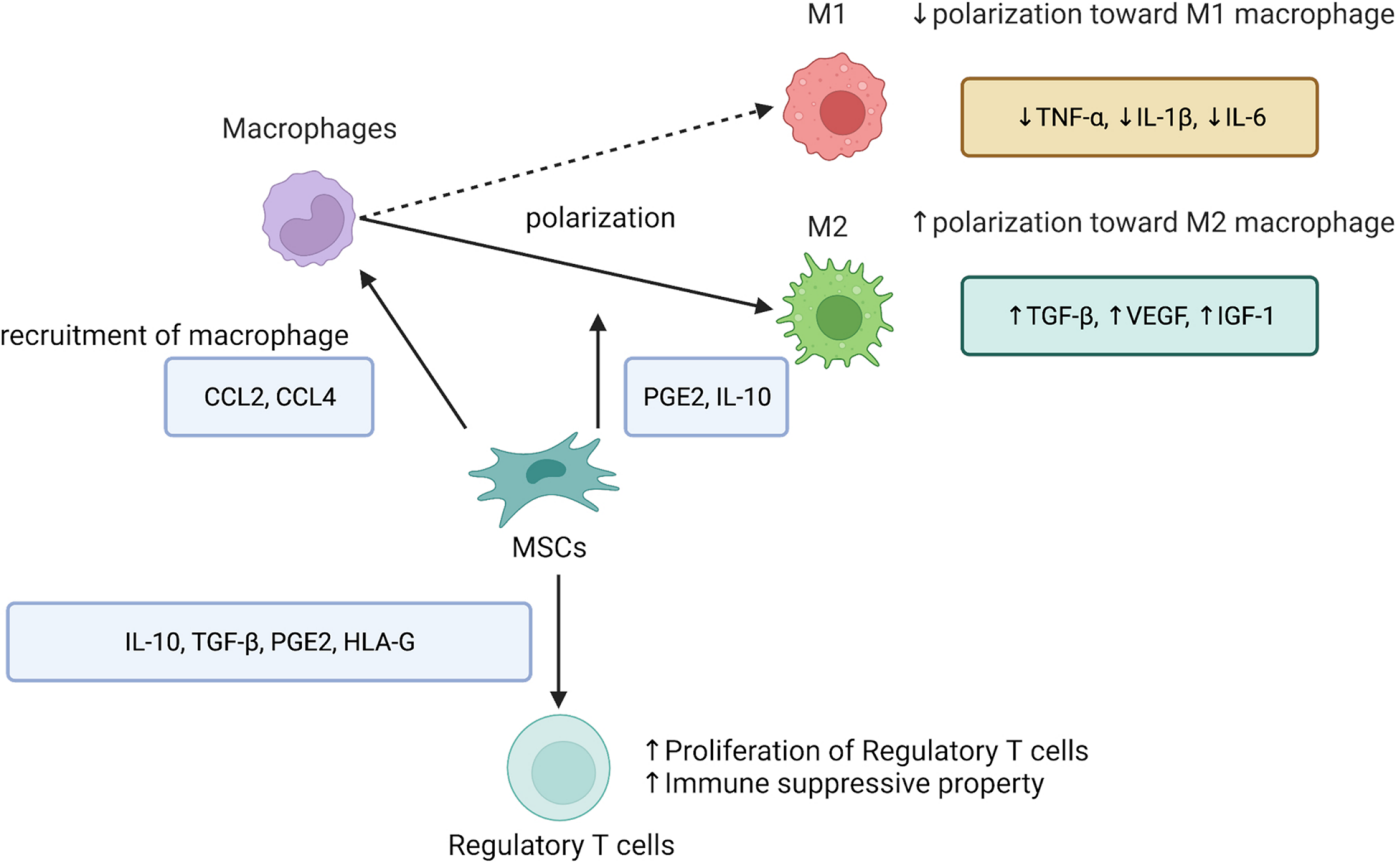
Immunomodulatory properties of MSCs. MSCs possess immunomodulatory properties that may benefit bone regeneration in inflammation: MSCs secret several chemokines, CCL2 and CCL4, potent chemoattractants for monocytes and macrophages. MSCs prevent M1 macrophage polarization and induce M2 polarization through cytokines such as PGE2 and Il-10 and then reduce the production of pro-inflammatory cytokines (TNF-α, IL-1β, and IL-6) from M1 macrophage and increased the secretion of growth factors (TGF-β, VEGF, and IGF-1) from M2 macrophages. MSCs can suppress adaptive immune responses by promoting the expansion and immune suppressive potency of regulatory T cells through the secretion of cytokines, such as IL-10, TGF-β, PGE2, and HLA-G

These findings suggest that MSCs regulate the chemotaxis and function of macrophages and that MSC-derived signals can contribute to bone regeneration by modulating macrophage function in inflammation (Fig. 2).

However, the effectiveness of MSC-based therapies may be influenced by chronic inflammation. The presence of chronic inflammation may inhibit the ability of MSCs to differentiate into osteoblasts and may also impair MSCs’ ability to promote the repair of damaged bone. Potential approaches to facilitating bone regeneration using immunomodulation are the preconditioning of MSCs, MSCs with anti-inflammatory cytokines, or exosomes to empower their immunomodulatory properties [86].

### Preconditioned MSCs with pro-inflammatory cytokines and hypoxia

Preconditioning MSCs with pro-inflammatory cytokines can affect MSCs’ secretory profile and osteogenic ability. IFN-γ-preconditioned MSCs upregulated indoleamine 2,3-dioxygenase (IDO), and the secretion of immunomodulatory molecules such as prostaglandin E2 (PGE2), hepatocyte growth factor (HGF), TGF-β, and CCL2 [86, 87]. TNF-α-preconditioned human adipose tissue-derived MSCs (AT-MSCs) promoted the proliferation and osteogenic differentiation of primary human osteoblastic cells [88]. IL-17A-preconditioned MSCs increased IL-6 and regulatory T-cell generation and inhibited Th1 cytokine secretion (TNF-α and IFN-γ) [89]. IL-17A promoted osteoblastic differentiation, inhibited adipogenic differentiation in MSCs, and accelerated osteoblastogenesis [90–93]. IL-6-preconditioned human adipose tissue-derived MSCs (AT-MSCs) demonstrated increased alkaline phosphatase (ALP) activity and mineralization [94, 95]. IL-8-preconditioned human AT-MSCs had reduced bone nodule formation but did not show changes in proliferation or osteogenic gene expression [94]. IL-17F-preconditioned human AT-MSCs had decreased proliferation yet enhanced ALP activity [94].

These studies suggest that pro-inflammatory cytokines and the species and tissue of origin may influence the osteogenic ability of preconditioned MSCs. However, there are few in vivo studies on the efficacy of preconditioned MSCs using pro-inflammatory cytokines; further research is needed in this area.

Hypoxia, or low oxygen levels, has several effects on MSCs. These effects can be relevant in the therapeutic use of MSCs, as the oxygen levels in some potential therapeutic situations are often lower than ambient levels (21% oxygen) [86, 87]. Hypoxia activates hypoxia-inducible factors (HIFs) that can increase MSC migration and bone healing [96]. MSCs cultured under hypoxic conditions have increased proliferation rates and secrete growth factors such as vascular endothelial growth factor (VEGF), bFGF, and platelet-derived growth factor-BB (PDGF-BB) [97–99]. In vivo studies of hypoxia preconditioned MSCs for bone healing are limited, yet some have shown improved collagen tissue formation, increased cell survival, and improved bone healing in mice and rats [100–102]. The molecular mechanisms of hypoxic conditioning on MSCs are not fully understood. However, these results suggest that the effects of hypoxia on MSCs can be translated to in vivo therapies, even in challenging situations such as fragility fractures in inflammaging.

### MSCs with local delivery of anti-inflammatory cytokines

Immunomodulation by the resolution of inflammation via local delivery of anti-inflammatory cytokines is a potential therapy for bone repair in inflammaging. Anti-inflammatory cytokines such as IL-4 and IL-13 can promote bone healing by accelerating the resolution of inflammation when applied locally; however, the effects are dependent on the timing and delivery method [103]. IL-4 and IL-13 can inhibit the proliferation of human osteoblasts but increase osteogenesis [103]. These cytokines polarize macrophages from an inflammatory M1 type to an anti-inflammatory M2 phenotype [104]. The interaction between MSCs and macrophages is essential for successful bone healing, and monoculture models may not accurately reflect these cytokines’ full immunomodulatory and osteogenic potential in vivo. In an MSC-macrophage co-culture model, adding IL-4 later increased calcified matrix formation and enhanced bone mineralization [28, 105]. Acute inflammation is necessary to initiate bone healing; however, resolving inflammation at the right time is critical for optimal bone formation.

A collagen scaffold containing IL-4 and IL-13 increased callus formation in a mouse bone defect model [22]. In a rat model, a decellularized bone matrix scaffold loaded with a low dose of IL-4 (10 ng) increased bone formation and vascularization, with favorable M1/M2 polarization ratios, when injected daily over the scaffold from 3 to 7 after surgery [106]. Higher doses of IL-4 or the matrix alone did not have the same effect. Other studies have used scaffolds that have sustained release of IL-4 or have a composite of microspheres releasing IL-4 to provide controlled, direct release of anti-inflammatory cytokines as a therapeutic strategy for improving bone healing [107, 108]. IL-4 prevented bone loss and accelerated bone formation by modulating local macrophage polarization to an M2 type in the murine chronic inflammatory femoral osteolysis model [109].

Genetically modified MSCs that secrete cytokines have been developed to provide controlled, direct-release cytokines. Lentivirus-transduced IL-4 over-expressing MSCs (IL4-MSCs) within microribbon scaffolds facilitated bone healing in aged murine long bone critical-size defect models by promoting polarization to an M2 macrophage phenotype [110]. To regulate the anti-inflammatory effect more precisely, NFκB-sensing-IL-4-secreting MSCs (NFκB-IL4-MSCs) were generated [111]. Elevated NFκB during chronic inflammation triggers NFκB-IL4-MSCs to secrete IL‐4; NFκB-IL4-MSCs only secrete IL‐4 during the ongoing inflammatory period, limiting potential adverse effects caused by excessive IL‐4 secretion [111]. NFκB-IL4-MSCs mitigated the pro-inflammatory response of macrophages exposed to wear particles by converting pro-inflammatory M1 to an anti-inflammatory M2 phenotype in vitro [112]. Local injections of NFκB-IL4-MSCs suppressed chronic inflammatory osteolysis, especially in female, in both young and aged mice by increasing the M2/M1 macrophage ratio [113, 114].

### Immunomodulatory effect of MSCs-derived exosome for bone regeneration

The indirect use of MSCs is gaining attention by exploiting the therapeutic potential of extracellular vesicles (EVs) derived from MSCs, as a means of overcoming some of the limitations of MSC therapy, such as the need for invasive procedures to obtain and administer MSCs, the risk of genetic instability and immunosuppression following allogeneic administration, and the difficulty in storing and transporting MSCs [115, 116]. MSC-derived EVs positively regulate osteogenic genes and osteoblastic differentiation without inhibiting proliferation in vitro [117]. The study also observed increased bone formation in critical-size calvarial bone defects in rats using an EVs delivery system, and identified miR-196a as a critical regulator of osteoblastic differentiation and osteogenic gene expression [117].

Exosomes are one type of EV produced by MSCs and are small (30–120 nm size), membrane-bound vesicles that contain proteins, lipids, and nucleic acids and serve as important mediators of intercellular communication [118]. MSCs produce large amounts of exosomes compared to other cells, which makes MSCs clinically viable for exosome separation and therapy [119]. Exosomes can be isolated using ultracentrifugation, density gradient centrifugation, and pegylation-based methods. Exosomes can be used for therapeutic purposes without the risk of genetic instability or immunosuppression following allogeneic administration in vivo [120]. Exosomes are easier to separate and store than MSCs, have lower immunogenicity, and are less likely to be trapped in the lungs or liver [120]. Exosomes can carry cytokines, chemokines, growth factors, enzymes, signaling molecules, miRNAs, lipids, and transcription factors and can have anti-inflammatory and anti-tumor effects and the ability to stimulate angiogenesis and enhance tissue repair and regeneration [120, 121].

MSC-derived exosomes improve bone regeneration by increasing osteogenic differentiation and angiogenesis [116]. MSC-derived exosomes inhibit apoptosis and promote the proliferation of osteoblasts and MALAT1-containing MSC-derived exosomes promote osteoblast differentiation through mediating microRNA-34c/SATB2 axis [122, 123]. In a rat model of femoral fracture, MSC-derived exosomes enhanced bone healing and angiogenesis [124]. In vitro, MSC-derived exosomes increased VEGF and HIF-1α expression and promoted osteogenic differentiation, as well as the proliferation, migration, and tube formation of human umbilical vein endothelial cells [124]. Induction of hypoxia leads to increased exosome production by MSCs, and these exosomes are more efficiently taken up by other MSCs [125]. Hypoxia also leads to increased expression of HIF-1α in MSCs, which is a significant factor in the positive regulation of miR-126 expression [125]. Thus, these exosomes contain large amounts of miR-126 and increase angiogenesis in endothelial cells by suppressing the expression of SPRED1 and activating the Ras/Erk signaling pathway [125]. In addition, miR-126 has been shown to promote angiogenesis during embryonic development by targeting PIK3R2, an inhibitor of angiogenic signals, and cell survival in response to VEGF [125].

Overall, using exosomes from MSCs may be a promising therapeutic approach for treating fragility fractures associated with inflammaging. However, the mechanisms by which exosomes promote these effects and their potential therapeutic effects in humans are not yet fully understood. Further research is needed to fully evaluate the safety and effectiveness of exosome therapy and determine the optimal sources, types, and doses of exosomes for this purpose. This will involve developing and testing exosome-based therapies in preclinical models and, eventually, in human clinical trials.

**Fig. 3:**
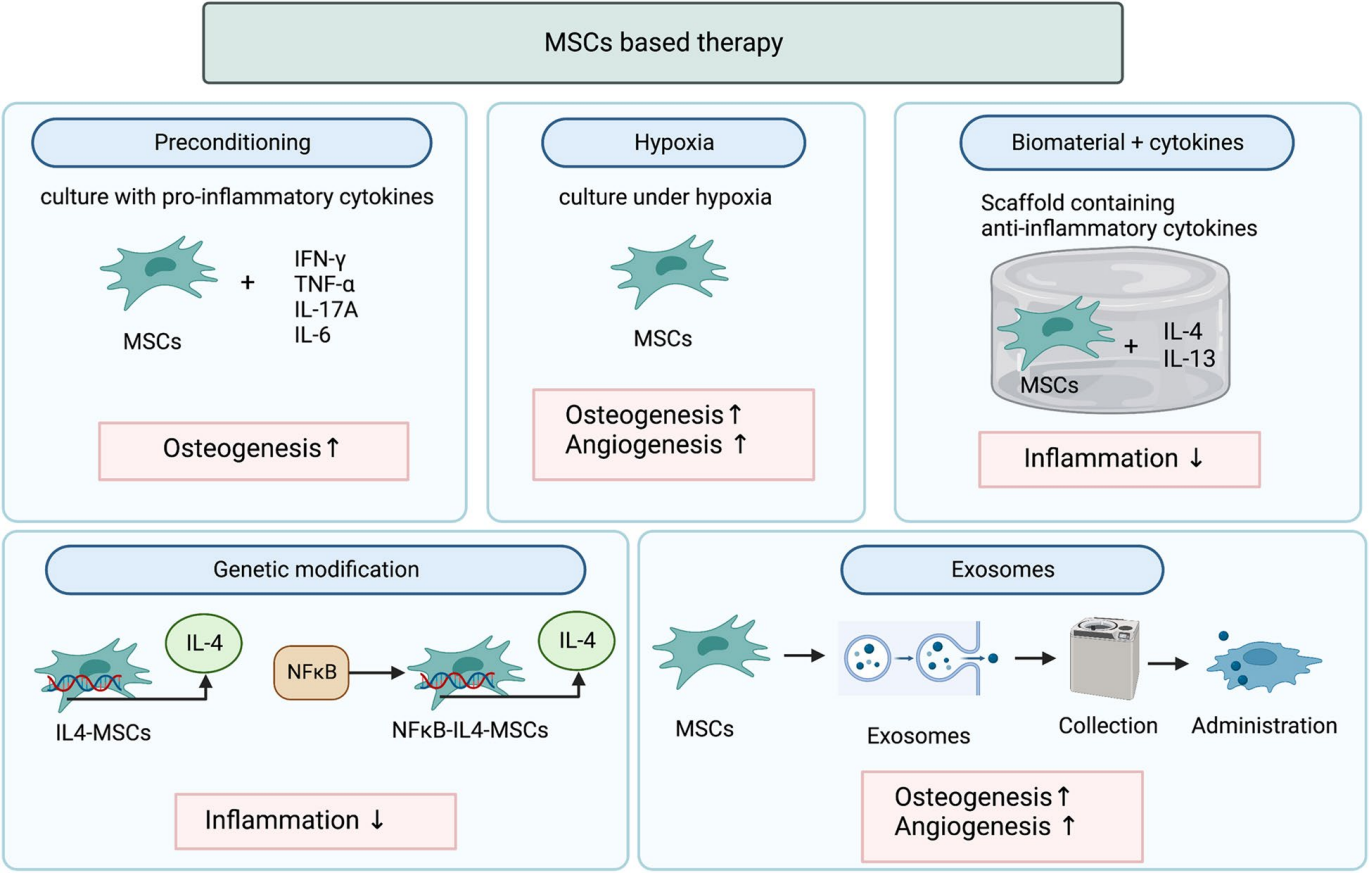
Future therapy for bone regeneration by modulating inflammation. Several methodologies can enhance the immunomodulatory and bone regenerative properties of MSCs. Preconditioning MSCs with pro-inflammatory cytokines (IFN-γ, TNF-α, IL-17A, IL-6) can affect MSCs’ secretory profile and osteogenic ability. MSCs cultured under hypoxic conditions increase osteogenesis and angiogenesis. Immunomodulation by resolving inflammation via local delivery of anti-inflammatory cytokines (IL-4, IL-13) is also a potential therapy for bone regeneration in inflammaging. Scaffolds containing anti-inflammatory cytokines and MSCs can resolve inflammation and enhance bone formation. Genetically modified MSCs that secrete anti-inflammatory cytokines suppress chronic inflammation and facilitate bone regeneration. MSC exosomes improve bone regeneration by increasing osteogenic differentiation, and angiogenesis

Figure 3 summarizes the future therapy for bone regeneration by modulating inflammation.

## Conclusions

The initiation of inflammation is crucial for bone healing after fracture. However, aging is associated with chronic inflammation (“inflammaging”), which can impair bone healing in the elderly and increase the risk of age-related diseases and functional decline. Macrophages show increased sensitivity and responsiveness to inflammatory signals with aging, leading to increased osteoclast activation and decreased osteoblast formation, resulting in increased bone resorption and decreased bone formation during healing.

Modulating inflammaging may be a promising approach for improving bone regeneration in the aging population. MSCs have immunomodulatory properties, modulate immune responses, and regulate macrophage chemotaxis; targeting macrophages and their activation through selective repolarization may also help promote bone healing.

MSCs can be preconditioned with pro-inflammatory cytokines or exposed to hypoxia to affect MSCs’ secretory profile and osteogenic ability. Preconditioning MSCs can modulate the immune response and polarize macrophages to an anti-inflammatory M2 phenotype, increasing MSCs migration and production of growth factors. Local delivery of anti-inflammatory cytokines can also modify the microenvironment and promote bone healing by accelerating the resolution of inflammation. MSCs produce large amounts of exosomes which can be used for therapeutic purposes without the risk of genetic instability or immunosuppression. Exosomes contain cytokines necessary for the bone repair process and stimulate the expression of genes associated with osteoblastic differentiation and angiogenesis.

These cell-based immunomodulatory therapies are also promising as a treatment for other chronic inflammatory diseases. Further research is needed to fully understand the mechanisms by which MSCs and macrophages interact in bone repair and to develop therapies that effectively suppress chronic inflammation and improve bone regeneration in the aging population.

## Data Availability

Not applicable.

## Abbreviations

PMNs: Polymorphonuclear neutrophils
IL-1: Interleukin-1
IL-6: Interleukin-6
TNF-α: Tumor necrosis factor-alpha
M-CSF: Macrophage colony-stimulating factor
iNOS: Inducible nitric oxide synthase
CCR2: CC chemokine receptor 2
TGF-β: Transforming growth factor-beta
IGF: Insulin-like growth factor
FGF: Fibroblast growth factor
PDGF: Platelet-derived growth factor
MIP-1: Macrophage inflammatory protein-1
MSCs: Mesenchymal stem cells
IFN-γ: Interferon-gamma
LPS: Lipopolysaccharides
IL-4: Interleukin-4
IL-13: Interleukin-13
SDF-1: Stromal cell-derived factor-1
CXCR4: C-X-C chemokine receptor 4
TLR4: Toll-like receptor 4
GM-CSF: Granulocyte-macrophage colony-stimulating factor
STAT5a: Signal transducer and activator of transcription 5a
MHC: Major histocompatibility complex
CFU: Colony-forming-unit
ALP: Alkaline phosphatase
SASP: Senescence-associated secretory phenotype
SA-β-gal: Senescence-associated β-galactosidase
NFκB: Nuclear factor kappa-light-chain-enhancer of activated B cells
PDPCs: Periosteum-derived progenitor cells
CCL2: C-C motif chemokine ligand 2
CCL4: C-C motif chemokine ligand 4
IDO: Indoleamine 2,3-dioxygenase
PGE2: Prostaglandin E2
HGF: Hepatocyte growth factor
HIFs: Hypoxia-inducible factors
VEGF: Vascular endothelial growth factor
PDGF-BB: Platelet-derived growth factor-BB
EVs: Extracellular vesicles
BMP: Bone morphogenetic proteins
